# Maternal and neonatal health service access and utilisation in sub-Saharan Africa (2015–2023): a systematic review and meta-analysis

**DOI:** 10.1186/s12913-026-14611-1

**Published:** 2026-04-27

**Authors:** Belete Achamyelew Ayele, Elizabeth Holliday, Tahir Ahmed Hassen, Catherine Chojenta

**Affiliations:** 1 Amhara Regional Health Bureau, Bahirdar, Amhara, Ethiopia; 2 School of Medicine and Public Health, College of Health, Medicine and Wellbeing, University of Newcastle, Newcastle, NSW Australia; 3 Centre for Women’s Health Research, College of Health, Medicine and Wellbeing, University of Newcastle, Newcastle, NSW Australia

**Keywords:** Maternal health, Neonatal health, Service utilisation, Sub-Saharan Africa

## Abstract

**Background:**

Despite maternal and neonatal health being crucial health indicators, in sub-Saharan Africa (SSA), access and utilisation remain low, leading to high maternal and neonatal death rates. Current epidemiological data is vital to inform policy planners to improve these health outcomes; however, relevant evidence from SSA is limited. This review assessed maternal (antenatal, delivery, and postnatal care) and neonatal health service use, access status, and associated factors in SSA. It aimed to raise awareness about the challenges mothers and newborns face in the region to support the development of sustainable solutions.

**Methods:**

Following Joanna Briggs Institute (JBI) guidelines, specific inclusion and exclusion criteria were established using the PECOS framework (Population, Exposure, Comparator, Outcome, and Study area). Medline, Embase, Web of Science, Emcare, CINAHL, Scopus, and Maternity and Infant Care databases were searched, and titles, abstracts, and full texts were reviewed to determine eligibility. Independent quality assessments were performed using JBI checklists. Data were extracted, and descriptive synthesis was performed. Random effects meta-analysis was used to estimate the proportion of deliveries occurring in a health facility and within SSA regions.

**Results:**

A total of 64 studies were included. Six studies focused exclusively on antenatal care (ANC), twenty on delivery care, eleven on maternal and neonatal postnatal care, and thirteen on mixed components of maternal health services. The proportion of mothers receiving at least one antenatal care (ANC) visit ranged from 22% in Chad to 99% in Ghana. The median prevalence of receiving four or more ANC visits across all SSA countries was 52.5%. Most women did not receive the World Health Organization’s recommended four or more ANC visits. Maternal delivery service utilisation varied widely across SSA countries due to financial constraints, transportation limitations, and low awareness of the importance of ANC services. The pooled proportion of deliveries conducted in a health facility was 63% (95% CI: 0.55, 0.72). In most countries, the utilisation of postnatal care services within the recommended 2–7 days was critically low. The practice of essential newborn care services, such as immediate breastfeeding initiation and skin-to-skin contact, were influenced by factors including caesarean section birth, ANC attendance, place and mode of delivery, maternal education, and awareness of neonatal danger signs.

**Conclusion and recommendations:**

In SSA, maternal and neonatal health service access and utilisation rates remain low and need more attention. Prioritisation of this issue by policymakers and healthcare providers, including the development of targeted interventions, is crucial to enhance maternal and neonatal healthcare access and utilisation.

**Supplementary information:**

The online version contains supplementary material available at 10.1186/s12913-026-14611-1.

## Background

Access to and utilisation of maternal and neonatal health services are crucial to reducing maternal and infant morbidity and mortality [1]. Universal and free access to health is a human right [2]; these services must be adequate, accessible, affordable, appropriate, and available [3]. Antenatal care (ANC) is vital for maternal and neonatal health outcomes and is recommended to start before 12 weeks gestation [4]. Maternal and neonatal health are interconnected from pregnancy to postnatal care, directly influencing the newborn’s health and development [5]. Newborn care ensures survival and well-being in the first 28 days of birth. Based on the World Health Organization (WHO) guidelines for postnatal care for the mother and newborn, postnatal care must address health vulnerabilities and continue at home or in a health facility after birth [6, 7].

Despite advances in medical technology and practices, global maternal and neonatal mortality and morbidity rates are high, largely due to inadequate access to quality care [8]. In sub-Saharan Africa (SSA), numerous countries have faced significant challenges in improving healthcare access and reducing mortality rates [9]. Globally, about 295,000 maternal deaths are recorded each year, primarily due to preventable and treatable causes such as bleeding, infection, hypertensive disorders of pregnancy, and abortion [10]. A reported 94% of these maternal deaths occur in low- and middle-income countries; SSA accounts for 70% of maternal [8, 11], and 42% of globally neonatal deaths equivalent with 27 deaths per 1000 live births [12]. According to a 2020 WHO report, approximately 2.4 million newborn infants died within the first month of life; SSA had the highest neonatal mortality rate in the world [13].

Challenges such as pandemics, limited healthcare infrastructure, and financial barriers had a significant impact on maternal and neonatal health [14, 15]. In areas with poor governance or conflict, these issues worsen, impacting maternal and neonatal survival [14, 16, 17]. In SSA, the lifetime maternal risk of mortality is one in thirty-eight women, which is five times the global average [8], and newborns have a tenfold higher risk of dying in the first month of life compared to those in high-income countries [15].

Despite efforts to improve maternal and neonatal health, utilisation rates in SSA are still low, and none of the SSA countries achieved the Millennium Development Goals (MDGs) targets for reducing maternal and neonatal deaths [18]. A study in eight SSA countries from 2018 to 2020 showed that more than 92% of pregnant women did not receive the recommended eight or more ANC contacts [19].

In SSA, a significant proportion of deliveries (an estimated 34% of deliveries) occur at home without skilled birth attendants [20]. Postnatal care (PNC) utilisation in SSA is also suboptimal, with about 48% of women not receiving at least one postnatal care visit between 2006 and 2018 [21]. Furthermore, coverage of newborn care services, such as the essential newborn practices of skin-to-skin contact and breastfeeding initiation is critically low [22, 23].

Despite international conventions advocating for equal health access globally, health inequality persists across regions [2, 24] and between countries [8]. COVID-19 also disproportionately affected maternal and neonatal healthcare in SSA [25, 26]. At this stage, the Sustainable Development Goals (SDGs [27], particularly Goal 3, which targets maternal and child health, are unlikely to be achieved. Goal 3.1 seeks to lower maternal mortality to under 70 per 100,000 live births, while Goal 3.2 aims to reduce neonatal mortality to 12 per 1,000 and under-five mortality to 25 per 1,000 by 2030 [27]. A literature review of ANC [19, 28–34], delivery, and postnatal care [20, 35–45], identified various determinants of maternal and neonatal health utilisation in SSA, summarised as individual, household, and community-level factors for low utilisations of these services.

Maternal and neonatal health services are development indicators affecting population health, economic productivity, and social progress [46]. Enhancing these services is essential for improving health outcomes and advancing global health initiatives. Even though current epidemiological data is vital to inform policy planners to improve maternal and neonatal health, information from the SSA region is limited.

Therefore, this study aimed to describe and examine the current utilisation status and determinant factors influencing maternal and neonatal health care coverage in SSA. This was achieved through systematic reviews and meta-analyses of existing primary literature.

## Methods

### Study inclusion and exclusion criteria

Following the Joanna Briggs Institute (JBI) guidelines [47], specific inclusion and exclusion criteria were established for study selection. The PECOS (Population, Exposure, Comparator, Outcome, and Study area) framework was used to formulate the study as follows:

**Population**: women of reproductive age (15–49 years) and newborns. (from birth up to 28 days old)

**Exposure**: Mothers and newborns who have received maternal and neonatal health care services.

**Comparator**: Depending on where the service was delivered, mothers and neonates who received health care at health facilities were compared to mothers and neonates who received health care through the mobility of the outreach service area or home health service.

**Outcome**: Level of maternal and or neonatal health care access and utilisation, and determinant factors.

**Study area:** Any SSA country or region (province). (S)

All observational or quantitative studies were included, regardless of their designs, if they examined access to and utilisation of maternal and neonatal health services. The inclusion criteria were as follows: the study should include maternal and/or neonatal individuals who received at least one ANC visit, delivery care, and/or at least one PNC and/or essential newborn care (ENBC) service within the study period; the article should be a primary study; the search was limited to the English language only; articles should be peer-reviewed and published between September 2015 and January 2023 (since the launch of the SDGs program); the study area must be within SSA countries or provinces. Case reports, case series, grey literature, conference abstracts, articles with unavailable full text, and animal studies were excluded.

The review was registered on the International Prospective Register of Systematic Reviews (PROSPERO) with the identifier CRD42023420656 in 2023. It is available at https://www.crd.york.ac.uk/prospero/display_record.php?ID=CRD42023420656.

### Search strategy

This review employed a two-step search strategy [48]. Initially, a preliminary search for studies using two sample databases, Medline and Embase, was conducted. In the second step, the search strategy was replicated across all databases, including Medline, Embase, Web of Science, Emcare, CINAHL, Scopus, and Maternity and Infant Care. We utilised all keywords and index terms identified in the first step for studies published in English between January 2015 and January 2023. The search strategy focused on four main concepts using Boolean operators. These included [1]: population terms like women, pregnant women, mothers, and neonate/newborn/infant [2]; service terms such as antenatal or prenatal care, skilled birth attendance or health facility delivery, intrapartum care, postnatal care, and essential newborn care like early initiation of breastfeeding and skin-to-skin contact [3]; access and utilisation terms, including access*, accessibility, affordability, utilis*, coverage, determinants or associated factors, and barriers; and [4] geography terms, which were the names of individual SSA countries. Both controlled vocabulary and free-text terms were used. Synonyms were joined with OR, and the main concept groups were joined with AND. Truncation and proximity operators were used when available. Full search strings, limits, and dates for each database are listed in Additional file 1.

### Study selection procedures and quality assessment

**Fig. 1:**
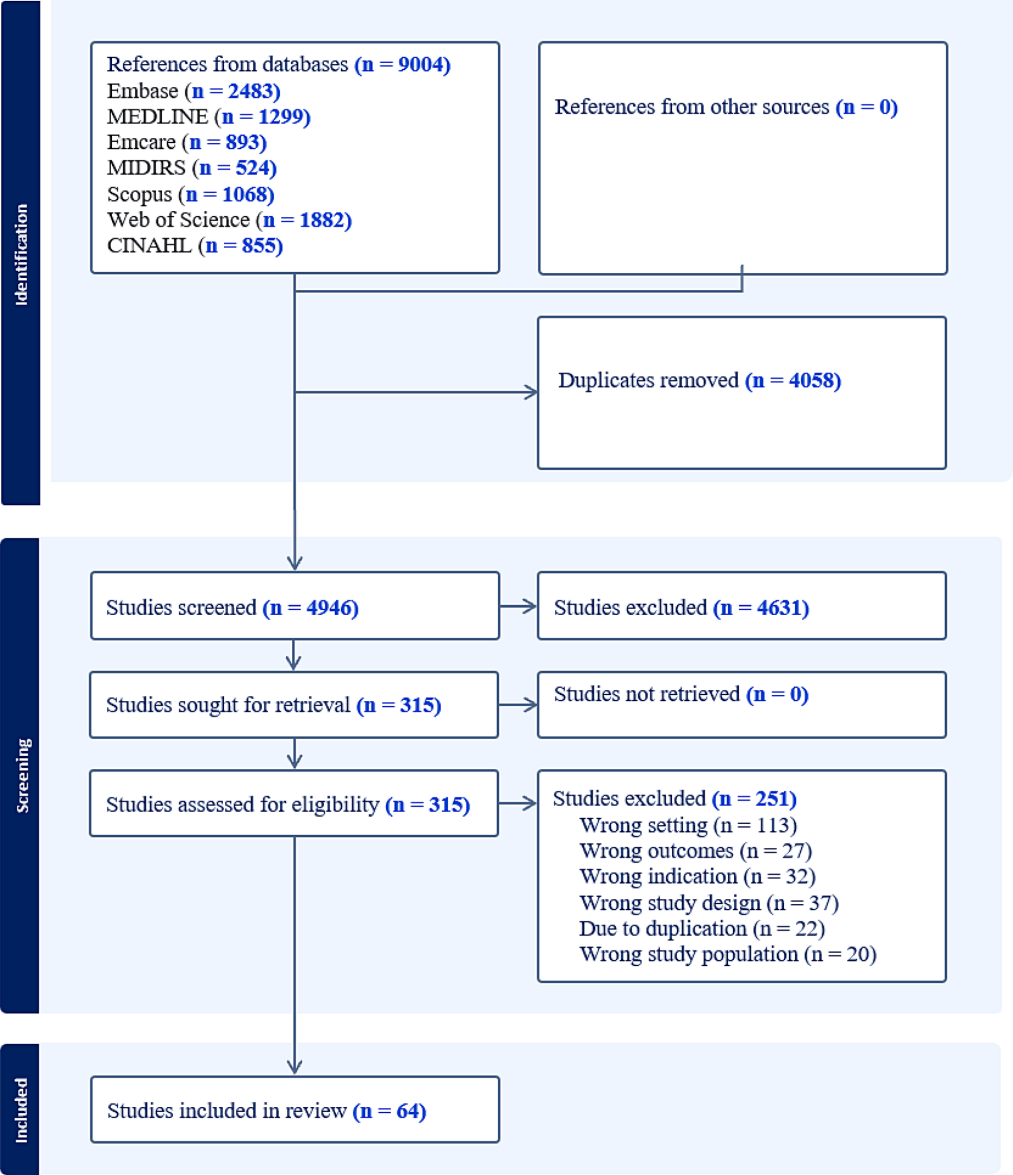
PRISMA flow diagram

Two screening levels were conducted, involving a title and abstract review, followed by full-text screening using EndNote 20 [49] and covidence for data management [50]. Two reviewers (BAA & TAH) independently screened the titles and abstracts of articles for eligibility and subsequently assessed each article in a full-text review. Disagreements were resolved through discussion. At title/abstract screening, records were excluded if they were clearly outside SSA countries, did not address maternal or neonatal health service access and/or utilisation outcomes, or did not meet the pre-specified eligibility criteria. At full-text screening, studies were excluded if they did not meet the eligibility criteria or did not report relevant data on maternal and neonatal service access or utilisation in SSA; reasons for full-text exclusions are summarised in Fig. 1. Study identification, screening, eligibility assessment, and inclusion were reported in accordance with the Preferred Reporting Items for Systematic Reviews and Meta-Analyses (PRISMA) guideline [51].

The methodological quality of included studies was independently assessed by two reviewers using the JBI critical appraisal checklists [52]. Critical appraisal involved evaluating the following aspects of the studies: sample frame appropriateness, sampling of study participants, sample size adequacy, detailed descriptions of study subjects and the setting, data analysis, method validity, standard measurement reliability, appropriateness of statistical analysis, and adequacy of the response rate.

### Data extraction

Data were extracted from included review articles using a data extraction format guided by JBI [53] and tailored to the specific research questions. One reviewer conducted the initial data extraction, and the second independently cross-checked the extracted data. Any disagreements were resolved through discussion.

For included studies, the following study characteristics were extracted: author’s name, year of publication, study design, study area (country), study period, sample size, study setting, maternal health services provided, neonatal health services provided, outcomes measured, main findings, prevalence rate of ANC, delivery, PNC and ENBC, study quality rating. Additionally, we collected information on independent variables that could impact the outcome of interest. See Additional File 2, which describes the characteristics of each study.

### Data synthesis and analysis

Descriptive statistics were used to describe the general characteristics of the studies. Findings on access to and utilisation of maternal and neonatal health services were reported through narrative descriptions, tables, and graphical summaries. Random effects meta-analysis was used to estimate a pooled estimate of the prevalence of health facility delivery. Heterogeneity was quantified using the I^2^ statistic, which determined the extent to which outcome variations were due to differences between studies rather than random chance [54]. A higher I^2^ value indicates more significant heterogeneity among the studies, suggesting that the variation in results is more likely due to differences rather than chance. Conversely, a lower I^2^ value suggests that the observed variation is more likely due to random variability and between-study variance (τ^2^) was using a DerSimonian and Laird random effects model [54]. The presence of publication bias in the included studies was assessed using funnel plots. A subgroup analysis was only performed for regions that contributed at least three studies [55]. Statistical analyses were conducted using Stata SE version 17.

### Operational definitions

- **Maternal Health**: Women’s health before and throughout pregnancy, childbirth, and the postnatal period [56].
- **Neonatal Health**: The health of newborns from birth to 28 days of life [57].
- **Essential Newborn Care (ENBC)**: Evidence-based interventions and practices to ensure newborn infants’ health, well-being, and survival in the immediate postnatal period [58]. ENBC includes care components such as drying the baby, skin-to-skin contact with the mother, early initiation of breastfeeding, cord care, temperature regulation, infection prevention, and early identification and management of potential health issues [58, 59].
- **Skin-to-skin contact (SSC):** A component of ENBC practice where a newborn baby is placed naked on the bare chest or abdomen of a parent or caregiver immediately after birth for bonding and temperature regulation [60].
- **Early Initiation of Breastfeeding (EIBF)**: Breastfeeding initiation within the first hour of a newborn’s life [61].

## Results

Across all databases, 9,004 studies were identified.

After removing duplicates, 4,946 articles were screened, and 315 full-text studies were considered using a priori eligibility criteria. Following a thorough assessment of full-text articles, 64 studies were identified for further analysis and included in this systematic review. The PRISMA flow diagram [51] is shown in Fig. 1.

### Characteristics of included studies

The 64 studies included in the review originated from various sub-regions of SSA and were published from 2015– 2023.These studies were selected after screening 315 full-text articles, of which 251 were excluded for the following reasons: wrong setting (*n* = 113), wrong outcomes (*n* = 27), wrong indication (*n* = 32), wrong study design (*n* = 37), duplicates (*n* = 22), and wrong study population (*n* = 20) (Fig. 1). More than 48% of studies were conducted in East Africa [33, 39, 42, 43, 45, 62–86], 37% in West Africa [29, 31, 44, 87–106], 12.5% in Southern Africa [36, 37, 41, 107–111], and 1.6% in Central Africa [112]. A detailed summary of study characteristics (country/sub-region, design, setting, sample size, outcomes, and extracted indicators) is provided in Additional File 2.

The largest number of studies (10 studies, or 15.6% of the total) were conducted in Ethiopia [33, 63, 71, 73, 75–77, 81, 84, 85], followed by Tanzania (seven studies) [39, 62, 64, 67, 79, 83, 86], Ghana (five studies) [29, 31, 91, 104, 106] and Nigeria (four studies) [89, 95, 96, 103]. Uganda [42, 66, 78], Kenya [38, 80, 82], Zambia [37, 108, 109], and Gambia [88, 101, 105] each contributed three studies. Sierra Leone [28, 100], South Sudan [43, 68], Malawi [36, 110], Burkina Faso [90, 99], Benin [92, 93], and Rwanda [69, 70] each contributed two studies. Fourteen countries each contributed a single study: Liberia [98], Eritrea [45], Senegal [102], Mozambique [41], Guinea-Bissau [44], Guinea [97], Burundi [72], Sudan [68], Eswatini [107], Mali [94], Cameroon [87], Chad [98], South Africa [111], and Somalia [65].

Most studies employed a cross-sectional design. Concerning the study settings, approximately 86% of the studies took place at the community level, while 11% were conducted at health facilities, and the remaining 3% were in both settings. Three studies were explicitly conducted in rural areas [45, 82, 103]. Sample sizes varied considerably across studies, ranging from 175 participants in a study from Somalia [65] to 29,992 participants in a Nigerian study [96].

The included studies investigated multiple dimensions of access to and utilisation of maternal and neonatal health services in the relevant country. Six studies [28, 29, 31, 33, 80, 98] focused exclusively on ANC; nineteen studies [36–39, 41–45, 63, 64, 67, 78, 84, 85, 100–102, 105] concentrated solely on the delivery phase; eleven studies [66, 69, 70, 74, 76, 79, 89, 91, 108, 110, 111] specifically addressed PNC, and fourteen studies [62, 68, 72, 77, 82, 87, 90, 92, 94, 97, 99, 103, 107, 112] combined components of maternal and neonatal health services. Fourteen studies [65, 71, 73, 75, 81, 83, 86, 88, 93, 95, 96, 104, 106, 109] evaluated essential newborn care practices, including skin-to-skin contact and breastfeeding initiation.

The critical appraisal process identified that most studies adhered to rigorous standards, including ensuring appropriate sample selection and size, providing comprehensive descriptions of subjects and settings, conducting reliable data analysis, using validated methods, and achieving satisfactory response rates. However, many studies did not employ appropriate methods for statistical analysis, such as the numerator and denominator, and percentages with confidence intervals were not clearly reported. The overall quality of studies on PNC service utilisation was rated as low. Additional File 3 provides details on each study’s quality assessment.

### Antenatal care service

About twenty studies focused on antenatal care, including the first ANC visit, early initiation of ANC, optimal ANC service utilisation, access to services, and any notable trends. For additional information, see Additional File 2. The sample sizes across the included studies exhibited a wide range; the average size across the included studies was approximately 5430 participants. Approximately 66% of pregnant women in Liberia [98], 44.8% (95% CI: 43.1–45.7) of women in Sierra Leone [28], and 35% in Chad [98] initiated their first ANC visit within the first trimester. In Kenya [82], a lower percentage of pregnant women initiated early ANC, with only 14% reporting their first ANC visit before 12 weeks of gestation due to low access to transportation, rural residency, low educational attainment among women, and cultural beliefs; similarly, in Cameroon [87], 35% of women initiated their first visit before 12 weeks of pregnancy; determinants included factors related to women’s empowerment, socioeconomic status, demographics, and cultural influences.

First ANC visit service utilisation ranged from low coverage of 22% in Chad [112] to 96% in Kenya [80] and 99% in Ghana [31]. Despite high coverage (81% in Ghana) [31], the median proportion of women receiving four or more ANC visits in the SSA region was 52.5%, with only 15.6% of women receiving four or more ANC visits in Chad [112]. The studies revealed that barriers such as financial constraints, lack of transportation, and limited awareness of the importance of ANC significantly impacted the utilisation of these services consistently. However, women who received community-based education and support were more likely to utilize ANC services optimally. In Sierra Leone, one study found that only 22% of women reported having eight or more ANC contacts [28].

Studies that explicitly focused on rural communities revealed that, in Nigeria, 52% of women had received four or more ANC visits [103], while in rural areas of Kenya [82], approximately 62% had done so. The analysis of trends in ANC utilisation revealed improvements in pregnant women accessing care services over the past decade. For example, in Ghana, studies covering the period from 2006 to 2018 showed a notable upward trajectory in ANC utilisation rates. Specifically, the proportion of mothers receiving four or more visits slightly increased from 49.3% in 2006 to 50% in 2011 and further to 58.6% in 2017–2018 [29]. However, although the WHO recommendation changed to eight contacts in 2016, ANC coverage of 8+ was not reported in the included studies. At the same time, women in Burkina Faso received at least four ANC services, with trends ranging from 33.6% to 44% between 2010 and 2014 [90, 99]; the results indicated that women with higher levels of education, individuals covered by health insurance, and households with higher incomes were more likely to attend ANC visits.

Some studies have assessed spatial disparities in ANC services within various countries. For instance, in Ethiopia [33], the prevalence of ANC service utilisation was found to be dependent on the distance to the nearest service facility and the specific regions within the country. Each additional kilometre of distance to the nearest ANC facility was associated with a reduced prevalence of having at least four ANC visits during pregnancy. The region of residence was found to explain 20% of the variability in achieving four or more ANC visits. Similarly, in Kenya, the coverage of four or more ANC visits and the number of visits varied significantly across the country. The prevalence of ANC service utilisation ranged from just 17% in Mandera to more than 77% in Nakuru Town West and Ruiru sub-counties [80].

In Burundi, a woman’s occupation and household wealth positively correlated with the probability of seeking ANC services from a healthcare provider [72]. Other studies examined ANC services. In Sudan, approximately 72.5% of women received their first ANC services [68]. ANC coverage of four or more visits was reported as follows: around 45.6% in Mali [94], 61.1% in Benin [92], 58.2% in Tanzania [62], and 35.3% in Guinea [97].

### Maternal delivery services

There were twenty-eight studies that examined the rate of institutional and non-institutional deliveries and five studies that evaluated the involvement of skilled birth attendants during labour and delivery (See the details in Additional File 2). Among these studies, the proportion of health facility/institutional deliveries varied significantly, ranging from as low as 25% in Sudan [68] to as high as 93% in Benin [92]. Other countries also reported relatively high rates of facility deliveries, including 90% in Uganda [78], 89.6% in Eswatini [107], 89% in Burkina Faso [90], about 85% in Zambia [37], and 84% in Sierra Leone [100]. Conversely, seven studies [36, 44, 45, 63, 68, 82, 103] found less than half of deliveries occurred in healthcare facilities or institutions. A study in the southern highlands of Tanzania found that skilled birth attendants were approximately 81% [39].

These studies have identified several factors that influenced access to and utilisation of facility-based skilled delivery services. These factors included maternal age, region, household wealth index, educational status, and distance to health facilities. For example, in Ethiopia, the likelihood of delivering in a healthcare facility was 14 times higher for women who attended four or more ANC visits [77]; however, there was an inverse relationship between health facility delivery and certain factors such as rural residence (OR = 0.352), higher parity (OR = 0.374), and practicing traditional religion (OR = 0.559) among women.

**Fig. 2:**
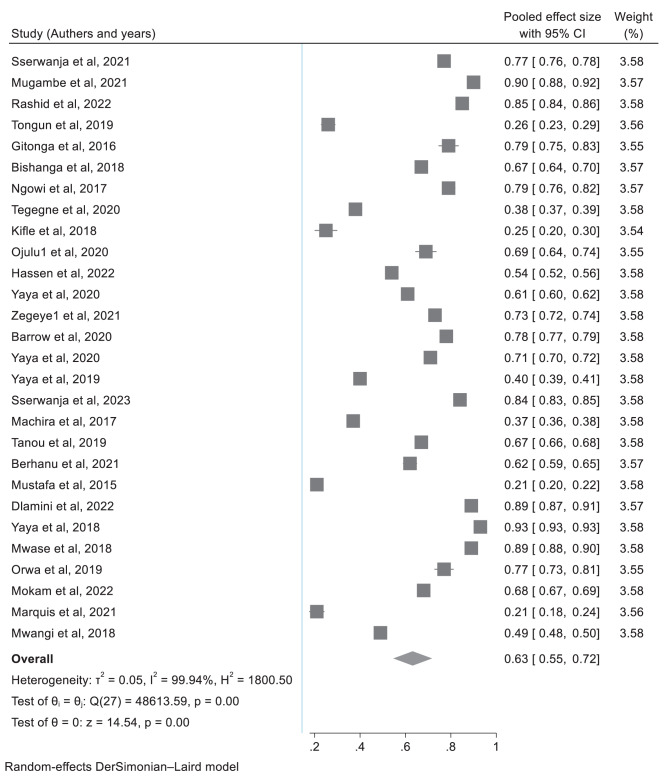
Overall pooled estimates of the prevalence of health facility delivery in sub-Saharan Africa

The random effects meta-analysis produced a pooled prevalence of health facility delivery of 63% (0.63; 95% CI: 0.55, 0.72). The prevalence of health facility maternal delivery varied widely among the studied countries, ranging from 21% in Sudan and Chad to 93% in Benin. Substantial heterogeneity across all the studies was observed (I^2^ = 99.94%, heterogeneity *p*-value < 0.01) (Fig. 2). When examined facility delivery by region, East Africa 57% (95% CI: 0.43, 0.70), Southern Africa 70% (95% CI: 0.43, 0.98), and West Africa 73% (95% CI: 0.63, 0.83) and within subgroups of regional variations and sample size, heterogeneity was still high. The funnel plot analysis revealed no evidence of publication bias. See Additional File 4 for details of the subgroup analysis and funnel plot outcomes.

### Postnatal care services

Nineteen studies assessed PNC service utilisation. Of these, 11 studies exclusively focused on PNC [66, 69, 70, 74, 76, 79, 89, 91, 108, 110, 111], while the remaining eight [62, 72, 82, 90, 92, 94, 107, 112] studies examined PNC in conjunction with ANC and delivery care. Details of how PNC was assessed across studies are provided in Additional File 2.

Four studies evaluated the early or immediate PNC [66, 69, 74, 108], while most others assessed care services up to 42 days (6 weeks) after delivery. Most studies assessed whether women received at least one PNC visit using binary yes/no questions. Most studies reported low service utilisation rates, except a few studies [66, 70, 91, 111] where a relatively high proportion was recorded.

In Ghana, where PNC coverage was 74%, access to health facilities was favoured for the use of PNC service [91]. In South Africa, a study was conducted to assess the status of PNC check-ups within the first 42 days after delivery. The findings revealed that approximately 60% of women received at least three PNC visits [66]. Similarly, in Uganda, trends in immediate PNC between 2001 and 2016 were evaluated [66], showing a 31% increase in PNC coverage; by 2016, 66% of women received postnatal care. Most initial checks occurred within 1 to 4 hours after childbirth; notably, the critical factors associated with receiving immediate postnatal care included women undergoing cesarean section births, with an Adjusted Odds Ratio (AOR) of 2.93 (95% CI: 2.28–3.75), and women who had received ANC with four or more visits with an AOR of 2.34 (95% CI: 1.50–3.64). Additionally, in Zambia, of 5,074 women, 63% (95% CI 61.9–64.6) received PNC within 48 hours after delivery; women who gave birth in a healthcare facility were more likely to utilize PNC than those who did not deliver in a health facility [108].

Despite nearly 99% of women in Nigeria reporting being aware of the benefits of PNC services, only 22% received these services [89]. In Sudan, access to early PNC services within 2–7 days after delivery at government health facilities was only 11.4% (AOR = 0.18; 95% CI: 0.05–0.61; *p* = 0.006). In Rwanda, a study examined various components of PNC, including cord care, measuring the baby’s temperature, providing counselling on newborn danger signs, offering breastfeeding counselling, and including an observed breastfeeding session. They found that approximately 44% (95% CI: 43–45.9) of participants received these services [70] and that factors such as radio exposure, visits by fieldworkers, and proximity to health facilities influenced the care provided. Another Rwandan study found that only 12.8% of individuals received PNC services within seven days of giving birth [69]. Only approximately 23.2% (95% CI: 18.7–28.3) of women who gave birth reported receiving postnatal care from a hospital, health centre, or dispensary in Tanzania [79].

Other studies conducted in various countries have also reported similarly low proportions of PNC services, such as 36.8% in Ethiopia [76], 48.4% in Malawi [110], 25.5% in Mali [110], 18.4% in Benin [92], 32.9% in Chad [112], and 38% in Kenya [82].

### Neonatal care service utilisation

In this review, essential newborn care mainly encompassed mother-to-child skin-to-skin contact (SSC), breastfeeding initiation, and other neonatal postnatal care practice. Extracted neonatal care indicators are summarised in Additional File 2. Out of the 14 studies included, approximately 28.6% were conducted in Ethiopia [71, 73, 75, 81]. Nigeria [95, 96], Ghana [88, 104], and Tanzania [83, 86] each accounted for 14.3% of the studies, with the remaining studies conducted in Gambia [88], Zambia [109], Benin [93], and Somalia [65] at various times related to neonatal health services utilisation.

In Ethiopia, one study revealed that the early initiation of breastfeeding (EIBF) rate rose from 70.5% in 2005 to 72.7% in 2016 [71]. Despite the overall improvement during this phase, a decline was observed between 2005 and 2011, dropping from 70.5% to 55.1% [71]. An increase in vaginal deliveries at health facilities contributed to the rise in early breastfeeding initiation rates across the surveys. A more recent study set in Ethiopia also estimated that the prevalence of EIBF was about 74% (with 95% CI71.65–75.47%) [73]. Early initiation of breastfeeding was 79% lower for those neonates delivered by caesarean section compared to neonates delivered vaginally (AOR = 0.21, CI: 0.13 to 0.33), whereas the odds of EIBF was nearly doubled for health facility-delivered neonates compared to neonates delivered at home (AOR = 1.98, 95% CI: 1.39 to 2.79). In Nigeria, the prevalence of EIBF varied; according to a 2018 survey, the EIBF rate was 43.8% [95], and several factors were associated with EIBF, including maternal age, maternal educational status, wealth index, multiparity (having multiple children), and facility delivery.

An assessment of essential newborn care services in Benin revealed that 55% of mothers undertook EIBF, 49% received breastfeeding support, and 47% received cord examination [93]. Ethnicity and caesarean delivery were significant independent variables across all surveyed areas.

Similarly, in Tanzania in 2015, 51% of neonates received EIBF [83]. A multivariate logistic regression analysis revealed that factors such as the place of delivery, mode of delivery, maternal educational status, and mothers’ knowledge about neonatal danger signs were positively and negatively associated with EIBF. In the same country, a 2020 study reported that 83% of respondents practised EIBF [86]. Among women surveyed, 94, 81, and 71% knew about colostrum giving, exclusive breastfeeding, and the recommended timing for initiating breastfeeding. In Ghana, 39.4% of newborns had breastfeeding initiated between one and six hours [104].

A study in Somalia assessed 175 women for essential newborn care practices. They found that the median post-birth facility stay was about 4 hours, only 8.6% (95% CI: 5.4–12.9) of newborns received immediate SSC services, and the early breastfeeding initiation prevalence was 30.1% (95% CI: 24.4 to 36.2) [65]. Almost all newborns experienced delayed first bathing until after 24 hours of birth.

In Ghana, approximately 25.5% (95% CI: 22.90–28.09) of newborns received recommended ENBC [106]. Public facilities had a higher prevalence of ENBC (26.4%) compared to private sector facilities (19.9%). Factors independently associated with good ENBC included cesarean section delivery (AOR = 0.33, 95% CI: 0.2–0.53) and the absence of health insurance coverage (AOR = 0.74, 95% CI: 0.56–0.97) [106]. This study defined good ENBC as encompassing early EIBF, SSC, and drying and wrapping of the baby. In addition, approximately 55 and 13% of newborn infants in Zambia [109] and Ethiopia [75] received neonatal care services, respectively.

One study identified that 35.7% of newborns in Gambia received SSC [88]. Factors found to be significant determinants of SSC included rural residence (AOR = 1.38, 95% CI: 1.06–1.79, *p* = 0.017), late ANC booking (AOR = 0.79, 95% CI: 0.68–0.93, *p* = 0.003), health facility delivery (AOR = 3.35, CI: 2.37–4.75, *p* < 0.001), and normal birth weight (AOR = 1.37, CI: 1.05–1.78, *p* = 0.019). In Nigeria, the prevalence of SSC was notably low, with only 12% of newborns receiving it [96]. Similarly, in Ethiopia, approximately 28% of mothers practised SSC on their newborns within the first hour of the postnatal period in health facilities [81].

## Discussion

SSA is a prominent region for research because maternal and neonatal healthcare access and service utilisation are low, reflecting the importance of addressing these gaps. This systematic review and meta-analysis comprehensively overviewed maternal and neonatal health services access and utilisation across SSA countries. The analysis encompassed various dimensions of care, including ANC, maternal delivery services, PNC services, and essential newborn care services, using many databases. Nearly half of the included studies were evaluated from East Africa, particularly Ethiopia, Tanzania, and Ghana, while Central Africa was under-represented. This imbalance is consistent with earlier reviews [34] and highlights ongoing geographic evidence gaps that may limit the representativeness of regional conclusions. Overall, the review indicates substantial disparities in service utilisation within and across countries in the region.

The findings regarding ANC services revealed that despite some countries reporting high coverage rates of one ANC visit, most women did not receive four or more ANC visits, and very few received the WHO-recommended eight or more ANC contacts [113]. This aligns with other assessments [19], which demonstrated that over 92% of pregnant women in SSA do not achieve ANC8+ contact. Indeed, this review found that ANC8+ contact coverage was rarely assessed; only one included study reported data explicitly on ANC8+ contact, indicating a critical evidence gap. In contrast, studies from South Asia have begun to report upward trends in ≥ 4 ANC visits [114], suggesting that SSA lags comparable low- and middle-income regions.

Early initiation of ANC and optimal ANC service utilisation are critical for maternal health, yet quantitative evidence indicates barriers related to access continue to affect specific regions; rural areas and underserved communities face challenges in ensuring timely and adequate antenatal care for pregnant women. Barriers such as financial constraints, transportation limitations, and awareness issues still need to be addressed; these findings align with other reports [115]. Furthermore, no studies were identified that specifically investigated the experiences of pregnant women who did not receive ANC services.

Substantial variation was also observed in facility-based deliveries across SSA, ranging from less than one-third of births in countries like Sudan and Chad to over 90% in Benin and Uganda. Our pooled prevalence estimate of 63% is slightly higher than earlier reviews, which reported averages closer to 60% [116], but it still falls significantly short of the global average of 84% [117]. Ghana, Uganda, Eswatini, Burkina Faso, Zambia, and Sierra Leone reported relatively high rates of health facility deliveries, reflecting their commitment to improving maternal health outcomes. However, many countries, such as Chad, Eritrea, Malawi, and Sudan, faced significant challenges in promoting facility-based childbirth.

In addition to facility-based deliveries, skilled birth attendants during labour and delivery were critical for ensuring safe childbirth, and their involvement varied across the included studies. The included studies identified several factors influencing access to and utilisation of facility-based skilled delivery services. These factors included maternal age, region, household wealth index, educational status, and distance to health facilities. The meta-analysis provided insight into the regional disparities in facility-based deliveries. The regional differences may be attributed to variations in healthcare infrastructure, cultural norms, and socioeconomic conditions [8]; this evidence was aligned with another previous study [30].

After synthesising findings from ANC, PNC, and delivery studies, we conducted a random-effects meta-analysis for the delivery pooled outcome since the data were sufficiently compatible and met the inclusion criteria.

In most studies, the JBI quality assessment tool indicated moderate to high methodological quality, although a few studies were rated as poor quality.

Substantial heterogeneity was observed across both the pooled and subgroup meta-analyses [54], likely due to variations in study design, setting, and sample size. Although the level of heterogeneity was high, the results of the sensitivity analyses conducted by sequentially excluding individual studies and low-quality articles did not alter the pooled estimates. This suggests that the heterogeneity primarily reflects differences in study contexts and methodologies rather than the influence of outliers.

Several countries reported poor access to early PNC services within 2–7 days, below the WHO recommendation [118]; challenges in promoting EINC IBF and SSC practices persisted in the studied areas.

The uptake of PNC within the neonatal period was particularly low in most countries, which is consistent with a previous evaluation that reported that nearly half of SSA women had no postnatal contact at all. By comparison, data from the WHO European region show that over 84% of women receive PNC visits, revealing a significant gap between SSA and other regions. This is especially concerning because the early postnatal period is the highest-risk window for maternal and neonatal complications and deaths.

Several factors significantly influenced maternal and neonatal PNC utilisation, including cesarean section births, receiving ANC with four or more visits, place of delivery, mode of delivery, maternal educational status, and mothers’ knowledge about neonatal danger signs.

### Clinical implications

The findings indicate that missed opportunities occur across the maternal–newborn continuum of care in SSA, especially in achieving recommended ANC contacts, accessing skilled facility delivery, and receiving early postnatal contact and essential newborn care. Clinically, low early PNC coverage is important because it occurs during the period of highest risk for maternal and neonatal complications and limits timely identification and management of problems, breastfeeding support, and counselling. The consistent association of lower utilisation with rural residence and socioeconomic disadvantage shows that inequities in access and service readiness are likely central drivers of preventable adverse outcomes.

## Conclusions

This systematic review and meta-analysis indicate that access to and utilisation of maternal and neonatal health services in SSA remain suboptimal and highly variable across countries and sub-regions. Evidence consistently shows gaps in achieving recommended ANC contacts, wide differences in facility-based delivery coverage, and generally low uptake of early postnatal care and essential newborn care practices. Service utilisation is patterned by socioeconomic and geographic inequalities, including maternal education, household wealth, and distance or access to facilities. Important evidence gaps remain, including limited assessment of ANC eight-contact coverage and under-representation of some sub-regions.

### Recommendations

Policy and programme: Make care more continuous from antenatal visits through delivery and early postnatal care by lowering costs, improving transport, and ensuring frontline facilities are ready and provide high-quality care. Expand early postnatal follow-up in both facilities and communities. Focus on strategies that support rural and low-income groups.

Research: Strengthen the evidence for eight or more antenatal visits and early PNC by conducting new, well-designed studies. Include more underrepresented regions and use qualitative research to learn why some women and newborns do not receive care.

### Strengths of the study

The study’s strengths include its comprehensive review of diverse SSA sub-regions, covering antenatal, delivery, postnatal, and essential newborn care. Employed robust systematic review and meta-analysis methods with geographical diversity offer credible insights into evolving healthcare practices, and its policy implications make it a valuable contribution to the region’s maternal and neonatal health services.

### Limitations of this study

Antenatal, postnatal, and neonatal care represent different maternal and newborn health stages, each requiring specific interventions and outcomes, making combining their results into one meta-analysis impractical and uneven distribution of studies. Heterogeneity across the included studies may limit comparability, highlighting the need for context-specific interpretation and locally tailored interventions. Additionally, the review’s limitation to quantitative studies may neglect insights that qualitative research provides. Finally, this review included only studies published in English, which may have caused the exclusion of relevant evidence from non-English publications.

## Electronic supplementary material

Below is the link to the electronic supplementary material.

Supplementary Material 1

Supplementary Material 2

Supplementary Material 3

Supplementary Material 4

## Data Availability

The datasets utilised in this study are attached as additional files. Furthermore, upon reasonable request, any other data also can be obtained from the corresponding author.

## Abbreviations

ANC: Antenatal care
EIBF: Early Initiation of Breastfeeding
ENBC: Essential Newborn care
JBI: Joanna Briggs Institute
MDG: Millennium Development Goals
PNC: Postnatal care
PRISMA: Preferred Reporting Item for Systematic Review and Meta-Analysis
SSC: Skin-to-Skin Contact
SSA: Sub-Saharan Africa
SDG: Sustainable Development Goals
WHO: World Health Organization
