# Supplementary material for: Maternal and neonatal health service access and utilisation in sub-Saharan Africa (2015–2023): a systematic review and meta-analysis
Source: BMC Health Serv Res. 2026 Apr 27;26:826. doi: 10.1186/s12913-026-14611-1 (PMC13270728; doi:10.1186/s12913-026-14611-1)
Supplement: Supplementary file 1 — Supplementary Material 1 [file 12913_2026_14611_MOESM1_ESM.docx]

**All databases’ Search History Supplementary materials**

**Embase Database Search History (2483 articles)**

--------------------------------------------------------------------------------

1 Women.mp. or Women/ (11735736)

2 Reproductive age women.mp. (3471)

3 Maternal.mp. (495839)

4 Maternal health.mp. or Maternal Health/ (26157)

5 Pregnant Women/ (103331)

6 Reproductive health.mp. or Reproductive Health/ (34609)

7 Mothers/ (106677)

8 Neonate.mp. (52999)

9 Newborn.mp. or Infant, Newborn/ (819277)

10 Infant/ (801452)

11 Neonatal health.mp. or Infant Health/ (32507)

12 p?ediatrics.mp. or Pediatrics/ (167154)

13 1 or 2 or 3 or 4 or 5 or 6 or 7 or 8 or 9 or 10 or 11 or 12 (12685680)

14 Antenatal care.mp. (16242)

15 Prenatal care.mp. or Prenatal Care/ (55483)

16 Antenatal follow-up.mp. (261)

17 Pregnancy care.mp. (1533)

18 Obstetrics/ or Obstetrics care.mp. (46043)

19 Midwifery/ or Delivery, Obstetric/ or Skilled birth attendance.mp. (49578)

20 Traditional births attendance.mp. (7)

21 Skilled delivery.mp. or Maternal Health Services/ (2789)

22 Intrapartum care.mp. (3390)

23 Parturition/ or Childbirth care.mp. (21803)

24 Labour.mp. (59059)

25 Midwi*.mp. (47949)

26 Perinatal services.mp. (265)

27 Perinatal care.mp. or Perinatal Care/ (17664)

28 Postnatal care.mp. or Postnatal Care/ (10152)

29 Postpartum care.mp. (175)

30 (Maternal adj3 services).mp. [mp=title, abstract, heading word, drug trade name, original title, device manufacturer, drug manufacturer, device trade name, keyword heading word, floating subheading word, candidate term word] (4205)

31 Essential newborn care.mp. (313)

32 Breast Feeding/ or Initiation of breastfeeding.mp. (66990)

33 Skin-to-skin contact.mp. or Kangaroo-Mother Care Method/ (2970)

34 Thermal care.mp. (202)

35 Newborn immunization.mp. (31)

36 Newborn immunization.mp. (31)

37 Infant care.mp. or Infant Care/ (3492)

38 14 or 15 or 16 or 17 or 18 or 19 or 20 or 21 or 22 or 23 or 24 or 25 or 26 or 27 or 28 or 29 or 30 or 31 or 32 or 33 or 34 or 35 or 36 or 37 (320851)

39 Health Facilities/ (80880)

40 Hospitals, Maternity/ or Hospitals, Public/ or Hospitals, District/ or Hospitals, Private/ or Hospitals, Community/ or Hospitals, Teaching/ or Hospitals, University/ or Hospitals, Pediatric/ or Hospitals, General/ or Hospitals/ or Hospitals, Rural/ or Hospitals, Urban/ or Hospitals, High-Volume/ (767893)

41 Mobile health units.mp. or Mobile Health Units/ (640)

42 Community Health Workers/ (9016)

43 Mobile health.mp. or Telemedicine/ (51413)

44 mHealth.mp. (8268)

45 Home Care Services/ or Home health service.mp. (60716)

46 Community health care.mp. or Community Health Services/ (57916)

47 Primary health care.mp. or Primary Health Care/ (93011)

48 39 or 40 or 41 or 42 or 43 or 44 or 45 or 46 or 47 (1085657)

49 Access.mp. (661323)

50 Health Services Accessibility/ or Accessibility.mp. (140014)

51 Affordability.mp. or "Costs and Cost Analysis"/ (69340)

52 Feasibility.mp. (370250)

53 Access to primary health care.mp. (374)

54 Catchment Area, Health/ or Hospital accessibility.mp. (273)

55 Utilis*.mp. (116578)

56 Coverage.mp. (202447)

57 Misuse.mp. (37516)

58 "Mills healthcare use ".mp. (0)

59 Challenges.mp. (504169)

60 Limitations.mp. (407421)

61 Determinants.mp. (243875)

62 Associated factors.mp. (41044)

63 Determinant factors.mp. (2202)

64 Factors.mp. (3503596)

65 Barriers.mp. (239630)

66 49 or 50 or 51 or 52 or 53 or 54 or 55 or 56 or 57 or 58 or 59 or 60 or 61 or 62 or 63 or 64 or 65 (5755876)

67 "Africa South of the Sahara"/ (17814)

68 Africa, Eastern/ (65026)

69 Africa, Central/ (1977)

70 Africa, Southern/ (67877)

71 Africa, Western/ (70843)

72 Angola/ (1858)

73 Benin/ (3215)

74 Botswana/ (3382)

75 Burkina Faso/ (5457)

76 Burundi/ (1113)

77 Cabo Verde/ (510)

78 Cameroon/ (8922)

79 Central Africa Republic.mp. (26)

80 Chad/ (1137)

81 Comoros/ (434)

82 Congo/ (4662)

83 "Democratic Republic of the Congo"/ or The Democratic Republic of Congo.mp. (7024)

84 Ivory coast.mp. or Cote d'Ivoire/ (4608)

85 Djibouti/ (427)

86 Equatorial Guinea/ (568)

87 Eritrea/ (725)

88 Eswatini/ (321)

89 Ethiopia/ (27013)

90 Gabon/ (2068)

91 Gambia/ (3275)

92 Ghana/ (15862)

93 Guinea/ (3272)

94 Guinea-Bissau/ (1263)

95 Kenya/ (26766)

96 Lesotho/ (972)

97 Liberia/ (2173)

98 Madagascar/ (5420)

99 Malawi/ (9669)

100 Mali/ (4218)

101 Mauritania/ (789)

102 Mauritius/ (1092)

103 Mozambique/ (4851)

104 Namibia/ (2043)

105 Niger/ (2969)

106 Nigeria/ (46943)

107 Rwanda/ (4846)

108 (Sao Tome and Principe).mp. [mp=title, abstract, heading word, drug trade name, original title, device manufacturer, drug manufacturer, device trade name, keyword heading word, floating subheading word, candidate term word] (270)

109 Senegal/ (8049)

110 Seychelles/ (563)

111 Sierra Leone/ (3105)

112 Somalia/ (2430)

113 South Africa/ (62458)

114 Sudan/ or South Sudan/ (9043)

115 Tanzania/ (19450)

116 Togo/ (1799)

117 Uganda/ (23308)

118 Zambia/ (8060)

119 Zimbabwe/ (8340)

120 67 or 68 or 69 or 70 or 71 or 72 or 73 or 74 or 75 or 76 or 77 or 78 or 79 or 80 or 81 or 82 or 83 or 84 or 85 or 86 or 87 or 88 or 89 or 90 or 91 or 92 or 93 or 94 or 95 or 96 or 97 or 98 or 99 or 100 or 101 or 102 or 103 or 104 or 105 or 106 or 107 or 108 or 109 or 110 or 111 or 112 or 113 or 114 or 115 or 116 or 117 or 118 or 119 (379695)

121 13 and 38 and 48 and 66 and 120 (3574)

122 limit 121 to (English language and yr="2015 -Current") (**2483)**

**Medline Database Search History (1299 articles)**

--------------------------------------------------------------------------------

1 Women.mp. or Women/ (1156057)

2 Reproductive-age women.mp. (2332)

3 Maternal.mp. (362755)

4 Maternal health.mp. or Maternal Health/ (26014)

5 Pregnant Women/ (13740)

6 Reproductive health.mp. or Reproductive Health/ (22325)

7 Mothers/ (54466)

8 Neonate.mp. (34705)

9 Newborn.mp. or Infant, Newborn/ (816244)

10 Infant/ (859950)

11 Neonatal health.mp. or Infant Health/ (2925)

12 p?ediatrics.mp. or Pediatrics/ (100967)

13 1 or 2 or 3 or 4 or 5 or 6 or 7 or 8 or 9 or 10 or 11 or 12 (2732933)

14 Antenatal care.mp. (12643)

15 Prenatal care.mp. or Prenatal Care/ (38697)

16 Antenatal follow-up.mp. (162)

17 Pregnancy care.mp. (1019)

18 Obstetrics/ or Obstetrics care.mp. (24504)

19 Midwifery/ or Delivery, Obstetric/ or Skilled birth attendance.mp. (51855)

20 Traditional birth attendance.mp. (5)

21 Skilled delivery.mp. or Maternal Health Services/ (16262)

22 Intrapartum care.mp. (972)

23 Parturition/ or Childbirth care.mp. (13634)

24 Labour.mp. (36685)

25 Midwi*.mp. (40282)

26 Perinatal services.mp. (210)

27 Perinatal care.mp. or Perinatal Care/ (7618)

28 Postnatal care.mp. or Postnatal Care/ (8309)

29 Post partum care.mp. (107)

30 (Maternal adj3 services).mp. [mp=title, book title, abstract, original title, name of substance word, subject heading word, floating sub-heading word, keyword heading word, organism supplementary concept word, protocol supplementary concept word, rare disease supplementary concept word, unique identifier, synonyms, population supplementary concept word, anatomy supplementary concept word] (19567)

31 Essential newborn care.mp. (259)

32 Breast Feeding/ or Initiation of breastfeeding.mp. (43512)

33 Skin-to-skin contact.mp. or Kangaroo-Mother Care Method/ (1710)

34 Thermal care.mp. (172)

35 Newborn immunization.mp. (29)

36 Newborn immunization.mp. (29)

37 Infant care.mp. or Infant Care/ (10855)

38 14 or 15 or 16 or 17 or 18 or 19 or 20 or 21 or 22 or 23 or 24 or 25 or 26 or 27 or 28 or 29 or 30 or 31 or 32 or 33 or 34 or 35 or 36 or 37 (236117)

39 Health Facilities/ (18698)

40 Hospitals, Maternity/ or Hospitals, Public/ or Hospitals, District/ or Hospitals, Private/ or Hospitals, Community/ or Hospitals, Teaching/ or Hospitals, University/ or Hospitals, Pediatric/ or Hospitals, General/ or Hospitals/ or Hospitals, Rural/ or Hospitals, Urban/ or Hospitals, High-Volume/ (223787)

41 Mobile health units.mp. or Mobile Health Units/ (3947)

42 Community Health Workers/ (6493)

43 Mobile health.mp. or Telemedicine/ (44916)

44 mHealth.mp. (8758)

45 Home Care Services/ or Home health service.mp. (36132)

46 Community health care.mp. or Community Health Services/ (34017)

47 Primary health care.mp. or Primary Health Care/ (107974)

48 39 or 40 or 41 or 42 or 43 or 44 or 45 or 46 or 47 (455564)

49 Access.mp. (419837)

50 Health Services Accessibility/ or Accessibility.mp. (139587)

51 Affordability.mp. or "Costs and Cost Analysis"/ (57729)

52 Feasibility.mp. (258782)

53 Access to primary health care.mp. (332)

54 Catchment Area, Health/ or Hospital accessibility.mp. (7290)

55 Utilis*.mp. (75100)

56 Coverage.mp. (160357)

57 Misuse.mp. (28838)

58 "Mills healthcare use ".mp. (0)

59 Challenges.mp. (408072)

60 Limitations.mp. (328803)

61 Determinants.mp. (193852)

62 Associated factors.mp. (33054)

63 Determinant factors.mp. (1698)

64 Factors.mp. (4958911)

65 Barriers.mp. (190508)

66 49 or 50 or 51 or 52 or 53 or 54 or 55 or 56 or 57 or 58 or 59 or 60 or 61 or 62 or 63 or 64 or 65 (6544405)

67 "Africa South of the Sahara"/ (13098)

68 Africa, Eastern/ (4370)

69 Africa, Central/ (1381)

70 Africa, Southern/ (2618)

71 Africa, Western/ (6286)

72 Angola/ (1127)

73 Benin/ (1889)

74 Botswana/ (2158)

75 Burkina Faso/ (3886)

76 Burundi/ (723)

77 Cabo Verde/ (248)

78 Cameroon/ (6430)

79 Central Africa Republic.mp. (15)

80 Chad/ (813)

81 Comoros/ (375)

82 Congo/ (2002)

83 "Democratic Republic of the Congo"/ or The Democratic Republic of Congo.mp. (5975)

84 Ivory coast.mp. or Cote d'Ivoire/ (4209)

85 Djibouti/ (250)

86 Equatorial Guinea/ (309)

87 Eritrea/ (410)

88 Eswatini/ (752)

89 Ethiopia/ (18556)

90 Gabon/ (1619)

91 Gambia/ (2662)

92 Ghana/ (10863)

93 Guinea/ (1282)

94 Guinea-Bissau/ (1021)

95 Kenya/ (19139)

96 Lesotho/ (514)

97 Liberia/ (1370)

98 Madagascar/ (3873)

99 Malawi/ (6602)

100 Mali/ (2663)

101 Mauritania/ (495)

102 Mauritius/ (622)

103 Mozambique/ (2973)

104 Namibia/ (1289)

105 Niger/ (1389)

106 Nigeria/ (33478)

107 Rwanda/ (3072)

108 (Sao Tome and Principe).mp. [mp=title, book title, abstract, original title, name of substance word, subject heading word, floating sub-heading word, keyword heading word, organism supplementary concept word, protocol supplementary concept word, rare disease supplementary concept word, unique identifier, synonyms, population supplementary concept word, anatomy supplementary concept word] (203)

109 Senegal/ (6182)

110 Seychelles/ (444)

111 Sierra Leone/ (1905)

112 Somalia/ (1853)

113 South Africa/ (48754)

114 Sudan/ or South Sudan/ (5375)

115 Tanzania/ (13595)

116 Togo/ (1269)

117 Uganda/ (14989)

118 Zambia/ (5411)

119 Zimbabwe/ (6512)

120 67 or 68 or 69 or 70 or 71 or 72 or 73 or 74 or 75 or 76 or 77 or 78 or 79 or 80 or 81 or 82 or 83 or 84 or 85 or 86 or 87 or 88 or 89 or 90 or 91 or 92 or 93 or 94 or 95 or 96 or 97 or 98 or 99 or 100 or 101 or 102 or 103 or 104 or 105 or 106 or 107 or 108 or 109 or 110 or 111 or 112 or 113 or 114 or 115 or 116 or 117 or 118 or 119 (255765)

121 13 and 38 and 48 and 66 and 120 (2294)

122 limit 121 to (english language and yr="2015 -Current") (1299)

**Emcare (Nursing and Allied Health) database Search History (893 articles)**

1 Women.mp. or Women/ (2020915)

2 Reproductive age women.mp. (1050)

3 Maternal.mp. (131881)

4 Maternal health.mp. or Maternal Health/ (10221)

5 Pregnant Women/ (31393)

6 Reproductive health.mp. or Reproductive Health/ (14268)

7 Mothers/ (55680)

8 Neonate.mp. (12599)

9 Newborn.mp. or Infant, Newborn/ (108072)

10 Infant/ (122464)

11 Neonatal health.mp. or Infant Health/ (8655)

12 p?ediatrics.mp. or Pediatrics/ (50792)

13 1 or 2 or 3 or 4 or 5 or 6 or 7 or 8 or 9 or 10 or 11 or 12 (2198865)

14 Antenatal care.mp. (7754)

15 Prenatal care.mp. or Prenatal Care/ (16965)

16 Antenatal follow-up.mp. (78)

17 Pregnancy care.mp. (682)

18 Obstetrics/ or Obstetrics care.mp. (8991)

19 Midwifery/ or Delivery, Obstetric/ or Skilled birth attendance.mp. (17414)

20 Traditional births attendance.mp. (5)

21 Skilled delivery.mp. or Maternal Health Services/ (632)

22 Intrapartum care.mp. (1391)

23 Parturition/ or Childbirth care.mp. (2889)

24 Labour.mp. (17175)

25 Midwi*.mp. (21908)

26 Perinatal services.mp. (131)

27 Perinatal care.mp. or Perinatal Care/ (3432)

28 Postnatal care.mp. or Postnatal Care/ (3270)

29 Post partum care.mp. (70)

30 (Maternal adj3 services).mp. [mp=title, abstract, heading word, drug trade name, original title, device manufacturer, drug manufacturer, device trade name, keyword heading word] (2014)

31 Essential newborn care.mp. (186)

32 Breast Feeding/ or Initiation of breastfeeding.mp. (18475)

33 Skin-to-skin contact.mp. or Kangaroo-Mother Care Method/ (1641)

34 Thermal care.mp. (117)

35 Newborn immunization.mp. (11)

36 Newborn immunization.mp. (11)

37 Infant care.mp. or Infant Care/ (1193)

38 14 or 15 or 16 or 17 or 18 or 19 or 20 or 21 or 22 or 23 or 24 or 25 or 26 or 27 or 28 or 29 or 30 or 31 or 32 or 33 or 34 or 35 or 36 or 37 (91721)

39 Health Facilities/ (23500)

40 Hospitals, Maternity/ or Hospitals, Public/ or Hospitals, District/ or Hospitals, Private/ or Hospitals, Community/ or Hospitals, Teaching/ or Hospitals, University/ or Hospitals, Pediatric/ or Hospitals, General/ or Hospitals/ or Hospitals, Rural/ or Hospitals, Urban/ or Hospitals, High-Volume/ (199502)

41 Mobile health units.mp. or Mobile Health Units/ (296)

42 Community Health Workers/ (3413)

43 Mobile health.mp. or Telemedicine/ (14782)

44 mHealth.mp. (4513)

45 Home Care Services/ or Home health service.mp. (26853)

46 Community health care.mp. or Community Health Services/ (20081)

47 Primary health care.mp. or Primary Health Care/ (32495)

48 39 or 40 or 41 or 42 or 43 or 44 or 45 or 46 or 47 (306050)

49 Access.mp. (215980)

50 Health Services Accessibility/ or Accessibility.mp. (49311)

51 Affordability.mp. or "Costs and Cost Analysis"/ (9314)

52 Feasibility.mp. (94138)

53 Access to primary health care.mp. (260)

54 Catchment Area, Health/ or Hospital accessibility.mp. (48)

55 Utilis*.mp. (31452)

56 Coverage.mp. (59753)

57 Misuse.mp. (15088)

58 "Mills healthcare use ".mp. (0)

59 Challenges.mp. (171136)

60 Limitations.mp. (117656)

61 Determinants.mp. (68316)

62 Associated factors.mp. (16430)

63 Determinant factors.mp. (765)

64 Factors.mp. (832105)

65 Barriers.mp. (104057)

66 49 or 50 or 51 or 52 or 53 or 54 or 55 or 56 or 57 or 58 or 59 or 60 or 61 or 62 or 63 or 64 or 65 (1486318)

67 "Africa South of the Sahara"/ (2843)

68 Africa, Eastern/ (13908)

69 Africa, Central/ (398)

70 Africa, Southern/ (13908)

71 Africa, Western/ (13908)

72 Angola/ (393)

73 Benin/ (763)

74 Botswana/ (949)

75 Burkina Faso/ (1295)

76 Burundi/ (255)

77 Cabo Verde/ (90)

78 Cameroon/ (1698)

79 Central Africa Republic.mp. (7)

80 Chad/ (214)

81 Comoros/ (65)

82 Congo/ (887)

83 "Democratic Republic of the Congo"/ or The Democratic Republic of Congo.mp. (1670)

84 Ivory coast.mp. or Cote d'Ivoire/ (739)

85 Djibouti/ (74)

86 Equatorial Guinea/ (92)

87 Eritrea/ (187)

88 Eswatini/ (110)

89 Ethiopia/ (6678)

90 Gabon/ (310)

91 Gambia/ (660)

92 Ghana/ (4738)

93 Guinea/ (553)

94 Guinea-Bissau/ (263)

95 Kenya/ (5800)

96 Lesotho/ (290)

97 Liberia/ (538)

98 Madagascar/ (738)

99 Malawi/ (2961)

100 Mali/ (764)

101 Mauritania/ (135)

102 Mauritius/ (257)

103 Mozambique/ (1225)

104 Namibia/ (572)

105 Niger/ (652)

106 Nigeria/ (9163)

107 Rwanda/ (1261)

108 (Sao Tome and Principe).mp. [mp=title, abstract, heading word, drug trade name, original title, device manufacturer, drug manufacturer, device trade name, keyword heading word] (58)

109 Senegal/ (1215)

110 Seychelles/ (133)

111 Sierra Leone/ (784)

112 Somalia/ (584)

113 South Africa/ (14688)

114 Sudan/ or South Sudan/ (1451)

115 Tanzania/ (4564)

116 Togo/ (411)

117 Uganda/ (5584)

118 Zambia/ (1992)

119 Zimbabwe/ (1806)

120 67 or 68 or 69 or 70 or 71 or 72 or 73 or 74 or 75 or 76 or 77 or 78 or 79 or 80 or 81 or 82 or 83 or 84 or 85 or 86 or 87 or 88 or 89 or 90 or 91 or 92 or 93 or 94 or 95 or 96 or 97 or 98 or 99 or 100 or 101 or 102 or 103 or 104 or 105 or 106 or 107 or 108 or 109 or 110 or 111 or 112 or 113 or 114 or 115 or 116 or 117 or 118 or 119 (81205)

121 13 and 38 and 48 and 66 and 120 (1297)

122 limit 121 to (English language and yr="2015 -Current") (893)

**Maternity & Infant Care Database (MIDIRS) database Search History (524 articles)**

--------------------------------------------------------------------------------

1 Women.mp. [mp=abstract, heading word, title] (117484)

2 Reproductive-age women.mp. [mp=abstract, heading word, title] (200)

3 Maternal.mp. [mp=abstract, heading word, title] (83259)

4 Pregnant women.mp. [mp=abstract, heading word, title] (32328)

5 Reproductive health.mp. [mp=abstract, heading word, title] (3661)

6 Mothers.mp. [mp=abstract, heading word, title] (38531)

7 Maternal health.mp. [mp=abstract, heading word, title] (14019)

8 Neonate.mp. [mp=abstract, heading word, title] (4191)

9 Newborn.mp. [mp=abstract, heading word, title] (39487)

10 Infant.mp. [mp=abstract, heading word, title] (79534)

11 Neonatal.mp. [mp=abstract, heading word, title] (49751)

12 Neonatal health.mp. [mp=abstract, heading word, title] (688)

13 Infant health.mp. [mp=abstract, heading word, title] (2447)

14 p?ediatrics.mp. [mp=abstract, heading word, title] (3668)

15 1 or 2 or 3 or 4 or 5 or 6 or 7 or 8 or 9 or 10 or 11 or 12 or 13 or 14 (228305)

16 Antenatal care.mp. [mp=abstract, heading word, title] (11038)

17 Prenatal care.mp. [mp=abstract, heading word, title] (4351)

18 Antenatal follow-up.mp. [mp=abstract, heading word, title] (46)

19 Pregnancy care.mp. [mp=abstract, heading word, title] (463)

20 Obstetrics care.mp. [mp=abstract, heading word, title] (24)

21 Skilled birth attendance.mp. [mp=abstract, heading word, title] (222)

22 Traditional birth attendance.mp. [mp=abstract, heading word, title] (1)

23 Skilled delivery.mp. [mp=abstract, heading word, title] (101)

24 Intrapartum care.mp. [mp=abstract, heading word, title] (3414)

25 Childbirth services.mp. [mp=abstract, heading word, title] (53)

26 Labour.mp. [mp=abstract, heading word, title] (29756)

27 Midwi*.mp. [mp=abstract, heading word, title] (39027)

28 Perinatal care.mp. [mp=abstract, heading word, title] (2089)

29 Postnatal care.mp. [mp=abstract, heading word, title] (5002)

30 Post-partum care.mp. [mp=abstract, heading word, title] (49)

31 Maternal health services.mp. [mp=abstract, heading word, title] (10149)

32 (Maternal adj3 services).mp. [mp=abstract, heading word, title] (10548)

33 Essential newborn care.mp. [mp=abstract, heading word, title] (124)

34 Initiation of breastfeeding.mp. [mp=abstract, heading word, title] (507)

35 Skin-to-skin contact.mp. [mp=abstract, heading word, title] (677)

36 Thermal care.mp. [mp=abstract, heading word, title] (80)

37 Newborn immunization.mp. [mp=abstract, heading word, title] (6)

38 Infant care.mp. [mp=abstract, heading word, title] (1682)

39 16 or 17 or 18 or 19 or 20 or 21 or 22 or 23 or 24 or 25 or 26 or 27 or 28 or 29 or 30 or 31 or 32 or 33 or 34 or 35 or 36 or 37 or 38 (83065)

40 Health facilities.mp. [mp=abstract, heading word, title] (1795)

41 Primary health care.mp. [mp=abstract, heading word, title] (1105)

42 Hospitals.mp. [mp=abstract, heading word, title] (11576)

43 Mobile health units.mp. [mp=abstract, heading word, title] (4)

44 Community health workers.mp. [mp=abstract, heading word, title] (393)

45 Mobile health.mp. [mp=abstract, heading word, title] (103)

46 mHealth.mp. [mp=abstract, heading word, title] (103)

47 Home health service.mp. [mp=abstract, heading word, title] (0)

48 Community health care.mp. [mp=abstract, heading word, title] (65)

49 40 or 41 or 42 or 43 or 44 or 45 or 46 or 47 or 48 (14598)

50 Access.mp. [mp=abstract, heading word, title] (9179)

51 Accessibility.mp. [mp=abstract, heading word, title] (1586)

52 Affordability.mp. [mp=abstract, heading word, title] (137)

53 Feasibility.mp. [mp=abstract, heading word, title] (2511)

54 Health service accessibility.mp. [mp=abstract, heading word, title] (6)

55 Access to primary health care.mp. [mp=abstract, heading word, title] (8)

56 Health care availability.mp. [mp=abstract, heading word, title] (4)

57 Hospital accessibility.mp. [mp=abstract, heading word, title] (3)

58 Utilis*.mp. [mp=abstract, heading word, title] (2605)

59 Coverage.mp. [mp=abstract, heading word, title] (3075)

60 Misuse.mp. [mp=abstract, heading word, title] (344)

61 Challenges.mp. [mp=abstract, heading word, title] (6411)

62 Limitations.mp. [mp=abstract, heading word, title] (3983)

63 Determinants.mp. [mp=abstract, heading word, title] (3449)

64 Associated factors.mp. [mp=abstract, heading word, title] (1087)

65 Determinant factors.mp. [mp=abstract, heading word, title] (59)

66 Factors.mp. [mp=abstract, heading word, title] (46410)

67 Barriers.mp. [mp=abstract, heading word, title] (5179)

68 50 or 51 or 52 or 53 or 54 or 55 or 56 or 57 or 58 or 59 or 60 or 61 or 62 or 63 or 64 or 65 or 66 or 67 (70728)

69 Africa South of the Sahara.mp. [mp=abstract, heading word, title] (0)

70 Africa, Eastern.mp. [mp=abstract, heading word, title] (5)

71 Africa, Central.mp. [mp=abstract, heading word, title] (5)

72 Africa, Southern.mp. [mp=abstract, heading word, title] (2)

73 Africa, Western.mp. [mp=abstract, heading word, title] (2)

74 Angola.mp. [mp=abstract, heading word, title] (55)

75 Benin.mp. [mp=abstract, heading word, title] (182)

76 Botswana.mp. [mp=abstract, heading word, title] (125)

77 Burkina Faso.mp. [mp=abstract, heading word, title] (316)

78 Burundi.mp. [mp=abstract, heading word, title] (53)

79 Cabo Verde.mp. [mp=abstract, heading word, title] (1)

80 Cameroon.mp. [mp=abstract, heading word, title] (206)

81 Central Africa Republic.mp. [mp=abstract, heading word, title] (0)

82 Chad.mp. [mp=abstract, heading word, title] (57)

83 Comoros.mp. [mp=abstract, heading word, title] (7)

84 Congo.mp. [mp=abstract, heading word, title] (293)

85 The Democratic Republic of Congo.mp. [mp=abstract, heading word, title] (164)

86 Cote d'Ivoire.mp. [mp=abstract, heading word, title] (95)

87 Djibouti.mp. [mp=abstract, heading word, title] (11)

88 Equatorial Guinea.mp. [mp=abstract, heading word, title] (5)

89 Eritrea.mp. [mp=abstract, heading word, title] (53)

90 Eswatini.mp. [mp=abstract, heading word, title] (9)

91 Ethiopia.mp. [mp=abstract, heading word, title] (1601)

92 Gabon.mp. [mp=abstract, heading word, title] (35)

93 Gambia.mp. [mp=abstract, heading word, title] (202)

94 Ghana.mp. [mp=abstract, heading word, title] (962)

95 Guinea.mp. [mp=abstract, heading word, title] (357)

96 Guinea-Bissau.mp. [mp=abstract, heading word, title] (108)

97 Kenya.mp. [mp=abstract, heading word, title] (1259)

98 Lesotho.mp. [mp=abstract, heading word, title] (41)

99 Liberia.mp. [mp=abstract, heading word, title] (88)

100 Madagascar.mp. [mp=abstract, heading word, title] (74)

101 Malawi.mp. [mp=abstract, heading word, title] (751)

102 Mali.mp. [mp=abstract, heading word, title] (160)

103 Mauritania.mp. [mp=abstract, heading word, title] (20)

104 Mauritius.mp. [mp=abstract, heading word, title] (15)

105 Mozambique.mp. [mp=abstract, heading word, title] (240)

106 Namibia.mp. [mp=abstract, heading word, title] (70)

107 Niger.mp. [mp=abstract, heading word, title] (133)

108 Nigeria.mp. [mp=abstract, heading word, title] (1840)

109 Rwanda.mp. [mp=abstract, heading word, title] (255)

110 (Sao Tome and Principe).mp. [mp=abstract, heading word, title] (5)

111 Senegal.mp. [mp=abstract, heading word, title] (211)

112 Seychelles.mp. [mp=abstract, heading word, title] (20)

113 Sierra Leone.mp. [mp=abstract, heading word, title] (207)

114 Somalia.mp. [mp=abstract, heading word, title] (122)

115 South Africa.mp. [mp=abstract, heading word, title] (1712)

116 South Sudan.mp. [mp=abstract, heading word, title] (54)

117 Sudan.mp. [mp=abstract, heading word, title] (299)

118 Tanzania.mp. [mp=abstract, heading word, title] (1080)

119 Togo.mp. [mp=abstract, heading word, title] (37)

120 Uganda.mp. [mp=abstract, heading word, title] (1003)

121 Zambia.mp. [mp=abstract, heading word, title] (532)

122 Zimbabwe.mp. [mp=abstract, heading word, title] (407)

123 69 or 70 or 71 or 72 or 73 or 74 or 75 or 76 or 77 or 78 or 79 or 80 or 81 or 82 or 83 or 84 or 85 or 86 or 87 or 88 or 89 or 90 or 91 or 92 or 93 or 94 or 95 or 96 or 97 or 98 or 99 or 100 or 101 or 102 or 103 or 104 or 105 or 106 or 107 or 108 or 109 or 110 or 111 or 112 or 113 or 114 or 115 or 116 or 117 or 118 or 119 or 120 or 121 or 122 (12869)

124 15 and 39 and 49 and 68 and 123 (765)

125 limit 124 to yr="2015 -Current" (524)

**Scopus Database Search History (1068 articles)**

(TITLE-ABS-KEY(Women OR "Reproductive-age women" OR Maternal OR "Pregnant women" OR "Reproductive health" OR Mothers OR "Maternal health" OR Neonate OR Newborn OR Neonatal OR "Neonatal health" OR "Infant health" OR p?ediatrics OR Infant) AND TITLE-ABS-KEY("Antenatal care" OR "Prenatal care" OR "Pregnancy follow-up" OR "Obstetric care" OR "Antenatal follow-up" OR "Skilled birth attendance" OR "Traditional birth attendance" OR "Childbirth care" OR "Skilled delivery" OR "Perinatal care" OR "Intrapartum care" OR "Maternal NEAR/5 services" OR "Reproductive health services" OR "Postnatal care" OR Midwi* OR "Post-partum care service" OR "Essential Newborn Care" OR "Initiation of breastfeeding" OR "exclusive breastfeeding" OR "Vitamin K prophylaxis" OR "Assessment of the newborn" OR "Skin-to-skin contact" OR "Thermal care" OR "Newborn immunization" OR "Newborn care service" OR "Infant care") AND TITLE-ABS-KEY("Health facilities" OR "Primary health care" OR Hospitals OR "Mobile Health Units" OR "Mobile health" OR mHealth OR "Home health service" OR "Community health worker" OR "Community health care" ) AND TITLE-ABS-KEY(Access OR Accessibility OR Affordability OR feasibility "Health service accessibility" OR "Access to primary health care" OR "Hospital accessibility" OR utili* OR Coverage OR Misuse OR "Health care use" OR Challenges OR Limitations OR Determinants OR "Associated factors" OR "Determinant factors" OR Factors OR Barriers) AND TITLE-ABS-KEY("Africa South of the Sahara" OR "Africa, Eastern" OR "Africa, Central" OR "Africa, Southern" OR "Africa, Western" OR Angola OR Benin OR Botswana OR "Burkina Faso" OR Burundi OR "Cabo Verde" OR Cameroon OR "Central Africa Republic" OR Chad OR Comoros OR Congo OR "The Democratic Republic of Congo" OR "Cote d'Ivoire" OR Djibouti OR "Equatorial Guinea" OR Eritrea OR Eswatini OR Ethiopia OR Gabon OR Gambia OR Ghana OR Guinea OR Guinea-Bissau OR Kenya OR Lesotho OR Liberia OR Madagascar OR Malawi OR Mali OR Mauritania OR Mauritius OR Mozambique OR Namibia OR Niger OR Nigeria OR Rwanda OR "Sao Tome And Principe" OR Senegal OR Seychelles OR "Sierra Leone" OR Somalia OR "South Africa" OR "South Sudan" OR Sudan OR Tanzania OR Togo OR Uganda OR Zambia OR Zimbabwe)) AND ( LIMIT-TO ( DOCTYPE,"ar" ) ) AND ( LIMIT-TO ( PUBYEAR,2023) OR LIMIT-TO ( PUBYEAR,2022) OR LIMIT-TO ( PUBYEAR,2021) OR LIMIT-TO ( PUBYEAR,2020) OR LIMIT-TO ( PUBYEAR,2019) OR LIMIT-TO ( PUBYEAR,2018) OR LIMIT-TO ( PUBYEAR,2017) OR LIMIT-TO ( PUBYEAR,2016) OR LIMIT-TO ( PUBYEAR,2015) ) AND ( LIMIT-TO ( LANGUAGE,"English" ) )

**Web of Science Final search history (1882 articles)**

(TS=(Women OR "Reproductive-age women" OR Maternal OR "Pregnant women" OR "Reproductive health" OR Mothers OR "Maternal health" OR Neonate OR Newborn OR Neonatal OR "Neonatal health" OR “Infant health" OR p?ediatrics OR Infant) AND TS=("Antenatal care" OR "Prenatal care" OR "Pregnancy follow-up" OR "Obstetrics care" OR "Antenatal follow-up" OR "Skilled birth attendance" OR "Traditional birth attendance" OR "Childbirth care" OR "Skilled delivery" OR "Perinatal care" OR "Intrapartum care" OR "Maternal NEAR/5 services" OR "Postnatal care" OR Midwi* OR "Postpartum care" OR "Essential Newborn Care" OR "Initiation of breastfeeding" OR "exclusive breastfeeding" OR "Vitamin K prophylaxis" OR "Assessment of the newborn" OR "Skin-to-skin contact" OR "Thermal care" OR "Newborn immunization" OR "Newborn care service" OR "Infant care") AND TS=("Health facilities" OR "Primary health care" OR Hospitals OR "Mobile Health Units" OR "Mobile health" OR mHealth OR "Home health service" OR "community health worker" OR "Community health care" ) AND TS=(Access OR Accessibility OR Affordability OR feasibility "Health service accessibility" OR "Access to primary health care" OR "Hospital accessibility" OR utili* OR Coverage OR Misuse OR "Health care use" OR Challenges OR Limitations OR Determinants OR "Associated factors" OR "Determinant factors" OR Factors OR Barriers) AND TS=("Africa South of the Sahara" OR "Africa, Eastern" OR "Africa, Central" OR "Africa, Southern" OR "Africa, Western" OR Angola OR Benin OR Botswana OR "Burkina Faso" OR Burundi OR "Cabo Verde" OR Cameroon OR "Central Africa Republic" OR Chad OR Comoros OR Congo OR "Democratic Republic of Congo" OR "Cote d’Ivoire" OR Djibouti OR "Equatorial Guinea" OR Eritrea OR Eswatini OR Ethiopia OR Gabon OR Gambia OR Ghana OR Guinea OR Guinea-Bissau OR Kenya OR Lesotho OR Liberia OR Madagascar OR Malawi OR Mali OR Mauritania OR Mauritius OR Mozambique OR Namibia OR Niger OR Nigeria OR Rwanda OR "Sao Tome And Principe" OR Senegal OR Seychelles OR "Sierra Leone" OR Somalia OR "South Africa" OR "South Sudan" OR Sudan OR Tanzania OR Togo OR Uganda OR Zambia OR Zimbabwe)) AND (LA==("ENGLISH") AND PY==("2023" OR "2022" OR "2021" OR "2020" OR "2019" OR "2018" OR "2017" OR "2016" OR "2015") AND DT==("ARTICLE"))=Total **1882**

| \|  \|  \| \| --- \| --- \| \|  \|  \| \|  \|  \| |
| --- | --- | --- | --- | --- | --- | --- |
|  |

**CINAHL (EBSCOhost) Databases Search History (855 articles)**

| \| **#** \| **Query** \| **Results** \|  \| \| --- \| --- \| --- \| --- \| \| S1 \| (MH "Women") \| 24,420 \|  \| \| S2 \| reproductive age women \| 4,870 \|  \| \| S3 \| (MH "Women's Health") \| 46,785 \|  \| \| S4 \| "Pregnant women" \| 43,919 \|  \| \| S5 \| (MH "Maternal-Child Health") OR "maternal health" \| 18,369 \|  \| \| S6 \| (MH "Reproductive Health") \| 9,413 \|  \| \| S7 \| neonate \| 28,466 \|  \| \| S8 \| (MH "Infant, Newborn") OR "newborns or neonates or infants" \| 147,451 \|  \| \| S9 \| Neonatal health \| 3,405 \|  \| \| S10 \| pediatrics \| 221,543 \|  \| \| S11 \| S1 OR S2 OR S3 OR S4 OR S5 OR S6 OR S7 OR S8 OR S9 OR S10 \| 487,175 \|  \| \| S12 \| (MH "Prenatal Care") OR "antenatal care" \| 22,594 \|  \| \| S13 \| Antenatal follow-up \| 195 \|  \| \| S14 \| (MH "Pregnancy Care (Saba CCC)") OR "Pregnancy care" \| 562 \|  \| \| S15 \| (MH "Obstetric Care") OR (MH "Delivery, Obstetric") OR (MH "Obstetric Service") OR (MH "Obstetric Nursing") \| 20,310 \|  \| \| S16 \| Skilled birth attendance \| 365 \|  \| \| S17 \| Traditional birth attendance \| 6 \|  \| \| S18 \| "skills delivery" \| 3 \|  \| \| S19 \| (MH "Intrapartum Care") OR "intrapartum care" \| 2,405 \|  \| \| S20 \| Midwi* \| 70,413 \|  \| \| S21 \| "childbirth care" \| 1,368 \|  \| \| S22 \| (MH "Perinatal Care") OR (MH "Perinatal Care (Saba CCC)") \| 5,296 \|  \| \| S23 \| (MH "Postnatal Care") OR (MH "Postpartum Care (Saba CCC)") OR "postnatal care or postpartum care" \| 6,386 \|  \| \| S24 \| (MH "Maternal Health Services") \| 11,853 \|  \| \| S25 \| "essential newborn care" OR (MH "Kangaroo Care") OR (MH "Intensive Care Units, Neonatal") OR (MH "Newborn Care (Saba CCC)") OR (MH "Infant Care") \| 22,032 \|  \| \| S26 \| (MH "Breast Feeding") OR (MH "Breast Feeding Promotion") OR "initiation of breastfeeding" \| 29,380 \|  \| \| S27 \| Thermal care \| 175 \|  \| \| S28 \| "newborn immunization" \| 6 \|  \| \| S29 \| S12 OR S13 OR S14 OR S15 OR S16 OR S17 OR S18 OR S19 OR S20 OR S21 OR S22 OR S23 OR S24 OR S25 OR S26 OR S27 OR S28 \| 162,902 \|  \| \| S30 \| (MH "Health Facilities") OR "health facilities" OR (MH "Hospitals, Public") \| 29,192 \|  \| \| S31 \| "hospitals or hospital" \| 354 \|  \| \| S32 \| (MH "Primary Health Care") OR (MH "Rural Health Personnel") OR (MH "Home Health Aides") \| 75,432 \|  \| \| S33 \| Primary health care unit \| 530 \|  \| \| S34 \| (MH "Home Health Aides") OR (MH "Rural Health Personnel") \| 2,259 \|  \| \| S35 \| (MH "Community Health Workers") OR (MH "Community Health Centers") OR (MH "Hospitals, Community") \| 17,130 \|  \| \| S36 \| (MH "Mobile Health Units") OR (MH "Telehealth") \| 15,938 \|  \| \| S37 \| S30 OR S31 OR S32 OR S33 OR S34 OR S35 OR S36 \| 133,992 \|  \| \| S38 \| "Access" \| 182,916 \|  \| \| S39 \| "accessibility" \| 117,888 \|  \| \| S40 \| Affordability \| 3,256 \|  \| \| S41 \| Feasibility \| 60,733 \|  \| \| S42 \| (MH "Health Services Accessibility") OR (MH "Health Services Needs and Demand") OR (MH "Health Services Misuse") \| 127,446 \|  \| \| S43 \| Hospital accessibility \| 164 \|  \| \| S44 \| Utilis* \| 26,641 \|  \| \| S45 \| Coverage \| 52,980 \|  \| \| S46 \| challenges \| 228,904 \|  \| \| S47 \| limitations \| 100,970 \|  \| \| S48 \| "determinants" \| 52,182 \|  \| \| S49 \| Associated factors \| 108,890 \|  \| \| S50 \| Determinant factors \| 2,561 \|  \| \| S51 \| Factors \| 1,848,180 \|  \| \| S52 \| Barriers \| 119,120 \|  \| \| S53 \| S38 OR S39 OR S40 OR S41 OR S42 OR S43 OR S44 OR S45 OR S46 OR S47 OR S48 OR S49 OR S50 OR S51 OR S52 \| 2,393,728 \|  \| \| S54 \| (MH "Africa South of the Sahara") \| 4,147 \|  \| \| S55 \| (MH "Africa, Eastern") OR (MH "Africa, Western") OR (MH "Africa, Southern") OR (MH "Africa, Central") \| 2,117 \|  \| \| S56 \| Angola \| 471 \|  \| \| S57 \| (MH "Angola") \| 297 \|  \| \| S58 \| (MH "Benin") \| 377 \|  \| \| S59 \| (MH "Botswana") \| 936 \|  \| \| S60 \| (MH "Burkina Faso") OR (MH "Cote d'Ivoire") \| 1,328 \|  \| \| S61 \| (MH "Burundi") \| 165 \|  \| \| S62 \| (MH "Cape Verde") \| 32 \|  \| \| S63 \| (MH "Cameroon") \| 1,432 \|  \| \| S64 \| (MH "Central African Republic") \| 117 \|  \| \| S65 \| (MH "Chad") \| 164 \|  \| \| S66 \| comoros \| 64 \|  \| \| S67 \| (MH "Congo") OR (MH "Democratic Republic of the Congo") \| 1,512 \|  \| \| S68 \| (MH "Djibouti") \| 58 \|  \| \| S69 \| (MH "Equatorial Guinea") OR (MH "Guinea") OR (MH "Guinea-Bissau") \| 572 \|  \| \| S70 \| (MH "Eritrea") \| 157 \|  \| \| S71 \| Eswatini \| 140 \|  \| \| S72 \| (MH "Ethiopia") \| 6,572 \|  \| \| S73 \| (MH "Gabon") \| 195 \|  \| \| S74 \| (MH "Gambia") \| 629 \|  \| \| S75 \| (MH "Ghana") \| 4,806 \|  \| \| S76 \| (MH "Kenya") \| 6,152 \|  \| \| S77 \| (MH "Lesotho") \| 214 \|  \| \| S78 \| (MH "Liberia") \| 597 \|  \| \| S79 \| (MH "Madagascar") \| 391 \|  \| \| S80 \| (MH "Malawi") \| 2,982 \|  \| \| S81 \| (MH "Mali") \| 598 \|  \| \| S82 \| (MH "Mauritania") \| 54 \|  \| \| S83 \| Mauritius \| 254 \|  \| \| S84 \| (MH "Mozambique") \| 957 \|  \| \| S85 \| (MH "Namibia") \| 407 \|  \| \| S86 \| (MH "Niger") \| 340 \|  \| \| S87 \| (MH "Nigeria") \| 9,296 \|  \| \| S88 \| (MH "Rwanda") \| 1,243 \|  \| \| S89 \| Sao Tome And Principe \| 23 \|  \| \| S90 \| (MH "Senegal") \| 704 \|  \| \| S91 \| Seychelles \| 140 \|  \| \| S92 \| (MH "Sierra Leone") \| 869 \|  \| \| S93 \| (MH "Somalia") \| 976 \|  \| \| S94 \| (MH "South Africa") \| 22,315 \|  \| \| S95 \| (MH "Sudan") OR "south sudan" \| 1,634 \|  \| \| S96 \| (MH "Tanzania") \| 4,410 \|  \| \| S97 \| (MH "Togo") \| 190 \|  \| \| S98 \| (MH "Uganda") \| 6,040 \|  \| \| S99 \| (MH "Zambia") \| 2,070 \|  \| \| S100 \| (MH "Zimbabwe") \| 1,784 \|  \| \| S101 \| S54 OR S55 OR S56 OR S57 OR S58 OR S59 OR S60 OR S61 OR S62 OR S63 OR S64 OR S65 OR S66 OR S67 OR S68 OR S69 OR S70 OR S71 OR S72 OR S73 OR S74 OR S75 OR S76 OR S77 OR S78 OR S79 OR S80 OR S81 OR S82 OR S83 OR S84 OR S85 OR S86 OR S87 OR S88 OR S89 OR S90 OR S91 OR S92 OR S93 OR S94 OR S95 OR S96 OR S97 OR S98 OR S99 OR S100 \| 84,350 \|  \| \| S102 \| S11 AND S29 AND S37 AND S53 AND S101 \| 1,020 \|  \| \| S103 \| S11 AND S29 AND S37 AND S53 AND S101 \| 856 \|  \| \| S104 \| S11 AND S29 AND S37 AND S53 AND S101 \| **855** \|  \| |
| --- | --- | --- | --- | --- | --- | --- | --- | --- | --- | --- | --- | --- | --- | --- | --- | --- | --- | --- | --- | --- | --- | --- | --- | --- | --- | --- | --- | --- | --- | --- | --- | --- | --- | --- | --- | --- | --- | --- | --- | --- | --- | --- | --- | --- | --- | --- | --- | --- | --- | --- | --- | --- | --- | --- | --- | --- | --- | --- | --- | --- | --- | --- | --- | --- | --- | --- | --- | --- | --- | --- | --- | --- | --- | --- | --- | --- | --- | --- | --- | --- | --- | --- | --- | --- | --- | --- | --- | --- | --- | --- | --- | --- | --- | --- | --- | --- | --- | --- | --- | --- | --- | --- | --- | --- | --- | --- | --- | --- | --- | --- | --- | --- | --- | --- | --- | --- | --- | --- | --- | --- | --- | --- | --- | --- | --- | --- | --- | --- | --- | --- | --- | --- | --- | --- | --- | --- | --- | --- | --- | --- | --- | --- | --- | --- | --- | --- | --- | --- | --- | --- | --- | --- | --- | --- | --- | --- | --- | --- | --- | --- | --- | --- | --- | --- | --- | --- | --- | --- | --- | --- | --- | --- | --- | --- | --- | --- | --- | --- | --- | --- | --- | --- | --- | --- | --- | --- | --- | --- | --- | --- | --- | --- | --- | --- | --- | --- | --- | --- | --- | --- | --- | --- | --- | --- | --- | --- | --- | --- | --- | --- | --- | --- | --- | --- | --- | --- | --- | --- | --- | --- | --- | --- | --- | --- | --- | --- | --- | --- | --- | --- | --- | --- | --- | --- | --- | --- | --- | --- | --- | --- | --- | --- | --- | --- | --- | --- | --- | --- | --- | --- | --- | --- | --- | --- | --- | --- | --- | --- | --- | --- | --- | --- | --- | --- | --- | --- | --- | --- | --- | --- | --- | --- | --- | --- | --- | --- | --- | --- | --- | --- | --- | --- | --- | --- | --- | --- | --- | --- | --- | --- | --- | --- | --- | --- | --- | --- | --- | --- | --- | --- | --- | --- | --- | --- | --- | --- | --- | --- | --- | --- | --- | --- | --- | --- | --- | --- | --- | --- | --- | --- | --- | --- | --- | --- | --- | --- | --- | --- | --- | --- | --- | --- | --- | --- | --- | --- | --- | --- | --- | --- | --- | --- | --- | --- | --- | --- | --- | --- | --- | --- | --- | --- | --- | --- | --- | --- | --- | --- | --- | --- | --- | --- | --- | --- | --- | --- | --- | --- | --- | --- | --- | --- | --- | --- | --- | --- | --- | --- | --- | --- | --- | --- | --- | --- | --- | --- | --- | --- | --- | --- | --- | --- | --- | --- | --- | --- | --- | --- | --- | --- | --- | --- | --- | --- | --- | --- | --- | --- | --- | --- | --- | --- | --- | --- | --- | --- | --- | --- | --- | --- |
