# Supplementary material for: Maternal and neonatal health service access and utilisation in sub-Saharan Africa (2015–2023): a systematic review and meta-analysis
Source: BMC Health Serv Res. 2026 Apr 27;26:826. doi: 10.1186/s12913-026-14611-1 (PMC13270728; doi:10.1186/s12913-026-14611-1)
Supplement: Supplementary file 4 — Supplementary Material 4 [file 12913_2026_14611_MOESM4_ESM.docx]

**

*Fig 3. Regional subgroup analysis of the prevalence of health facility delivery in sub-Saharan Africa*.

*Fig 4.* Sample size-based subgroup analysis of the prevalence of health facility delivery in sub-Saharan Africa*.*

*Figure 5: Funnel plot for sub-Saharan Africa prevalence of health facility delivery meta-analysis*
